# Molecular and Clinical Characterization of *CNGA3* and *CNGB3* Genes in Brazilian Patients Affected with Achromatopsia

**DOI:** 10.3390/genes14061296

**Published:** 2023-06-20

**Authors:** Rebeca A. S. Amaral, Fabiana L. Motta, Olivia A. Zin, Mariana M. da Palma, Gabriela D. Rodrigues, Juliana M. F. Sallum

**Affiliations:** 1 Department of Ophthamology, Federal University of São Paulo (UNIFESP), São Paulo 04023-062, Brazil; rebeca.azsouza@gmail.com (R.A.S.A.); olivia.zin@gmail.com (O.A.Z.); marimatioli@yahoo.com.br (M.M.d.P.); gabriela.dona@unifesp.br (G.D.R.); 2 Instituto de Genética Ocular, São Paulo 04552-050, Brazil; fabiana.louise@gmail.com; 3 Instituto Brasileiro de Oftalmologia (IBOL), Rio de Janeiro 22250-040, Brazil; 4 Department of Surgery & Hospital Clinic of Barcelona, School of Medicine, Universitat de Barcelona, 08007 Barcelona, Spain

**Keywords:** achromatopsia, cone photoreceptor, *CNGA3*, *CNGB3*

## Abstract

Achromatopsia (ACHM) is a congenital cone photoreceptor disorder characterized by reduced visual acuity, nystagmus, photophobia, and very poor or absent color vision. Pathogenic variants in six genes encoding proteins composing the cone phototransduction cascade (*CNGA3*, *CNGB3*, *PDE6C*, *PDE6H*, *GNAT2*) and of the unfolded protein response (*ATF6*) have been related to ACHM cases, while *CNGA3* and *CNGB3* alone are responsible for most cases. Herein, we provide a clinical and molecular overview of 42 Brazilian patients from 38 families affected with ACHM related to biallelic pathogenic variants in the *CNGA3* and *CNGB3* genes. Patients’ genotype and phenotype were retrospectively evaluated. The majority of *CNGA3* variants were missense, and the most prevalent *CNGB3* variant was c.1148delC (p.Thr383Ilefs*13), resulting in a frameshift and premature stop codon, which is compatible with previous publications in the literature. A novel variant c.1893T>A (p.Tyr631*) in the *CNGB3* gene is reported for the first time in this study. A great variability in morphologic findings was observed in our patients, although no consistent correlation with age and disease stage in OCT foveal morphology was found. The better understanding of the genetic variants landscape in the Brazilian population will help in the diagnosis of this disease.

## 1. Introduction

Achromatopsia (ACHM) is a rare genetic retinal disease that is inherited in an autosomal recessive; it is estimated to affect one in 30,000 people [1,2]. Clinical symptoms usually present after birth or early infancy, and are typically characterized by reduced visual acuity, nystagmus, photophobia, and very poor or absent color vision. These symptoms are due to a primary functional defect of the cone photoreceptors that is reflected in a severely reduced or absent light-adapted electroretinogram (ERG) and a preserved scotopic ERG signal. While the fundus appearance is often normal, abnormal foveal reflex, pigmentary mottling, and atrophic changes may be found in the macula area, especially in advanced cases [3,4]. Patients do not report progression of symptoms, and the disease was initially thought to be nonprogressive. However, previous studies established structural alterations and foveal findings that can emerge and are compatible with a slow progressive degeneration and loss of cone photoreceptor cells [5,6].

ACHM can be defined as complete or incomplete depending on the extent of cone photoreceptor dysfunction and resulting severity of symptoms [7]. Patients with incomplete ACHM present with a milder phenotype, residual color discrimination, better visual acuity, and/or residual photopic ERG responses [2,3]. In these cases, the diagnosis is occasionally made even later in childhood.

Pathogenic variants in six genes encoding components of the cone phototransduction cascade (*CNGA3*, *CNGB3*, *PDE6C*, *PDE6H*, *GNAT2*) and of the unfolded protein response (*ATF6*) account over 90% of ACHM cases, while *CNGA3* and *CNGB3* alone are responsible for most cases [1,2]. *CNGB3* pathogenic variants constitute approximately 40–50% of cases and are more common in the Caucasian population (Europe and the United States) [8,9,10]. *CNGA3* pathogenic variants underlie approximately 30–40% of cases and are more common in the Asian population (Middle East and China), accounting for 80% of all cases in this region [11].

The majority of *CNGB3* pathogenic variants are nonsense, frameshift, or splicing mutations that result in truncated or loss of function channel proteins [8,9,10,11]. In contrast, most *CNGA3* pathogenic variants are missense mutations that affect only single amino acid residues of the protein [6,11,12].

In 2018, our group published the frequency of inherited retinal dystrophies in Brazil [13]. At that time, we reported on six patients with ACHM-associated genes. Now, we are able to expand our study on *CNGA3* and *CNGB3* pathogenic variants related to ACHM in Brazilian patients.

## 2. Materials and Methods

### 2.1. Patient Selection

A retrospective study of medical records from two centers specializing in inherited retinal dystrophies was performed, one located in São Paulo (Federal University of São Paulo and Instituto de Genética Ocular) and one in Rio de Janeiro (Instituto Brasileiro de Oftalmologia). Forty-six Brazilian patients with clinical diagnosis of ACHM were identified. Among them, 42 patients from 38 families had conclusive genetic testing with pathogenic variants in *CNGA3* and *CNGB3* genes. Medical and family histories were collected, as were genetic data. Only patients in whom the diagnosis could be genetically confirmed were included.

### 2.2. Ophthalmic Examination

The clinical diagnosis of ACHM was based on detailed clinical examination, visual function, signs/symptoms, ophthalmologic features, and age of onset. Patients underwent detailed ophthalmic exams, including best-corrected visual acuity (BCVA), contrast sensitivity (CS), slit-lamp exams, and multimodal retinal imaging using color fundus images, fundus autofluorescence (FAF), and optical coherence tomography (OCT). BCVA was assessed with the Early Treatment Diabetic Retinopathy Study (ETDRS) chart, while CS was measured with the Pelli Robson chart at 1 m.

Fundus photographs were reviewed to confirm the findings reported in the medical record. 

The severity of degeneration shown on the OCT was graded in different stages: preserved inner segment ellipsoid, disrupted inner segment ellipsoid, inner segment ellipsoid loss, presence of a hyporeflective zone, inner segment, and retinal pigment epithelium (RPE) loss.

### 2.3. Genotyping

The records of patients who underwent genetic testing for ACHM causative variants in CNG genes were reviewed. Genes associated with ACHM were included in a larger panel of genes associated with inherited retinal disease. ACHM was considered genetically confirmed if two pathogenic or likely pathogenic variants in one of the six known genes were identified in the patient. Segregation was performed when possible.

Genetic analysis was performed using commercial next-generation sequence (NGS) panels for inherited retinal disorders with either 224 or 330 genes (see Supplemental Table S1 for the list of genes analyzed). Genomic DNA obtained from the submitted sample was enriched for targeted regions using a hybridization-based protocol and sequenced using Illumina technology. Confirmation of the presence and location of reportable variants was performed based on stringent criteria established by each accredited diagnostic laboratory.

The standards and guidelines provided by the American College of Medical Genetics and Genomics (ACMG) and the Association for Molecular Pathology (AMP) [14] were applied in order to classify the identified variants. Novel variants were classified as pathogenic or likely pathogenic when representing a loss of function variant (frameshift or nonsense or copy number variation or affecting a canonical splice site). In addition, pathogenic score was evaluated when allele frequency in the gnomAD population databases was extremely low. Two platforms were assessed that combine computational predictions with clinical support, segregation, or functional studies in order to assist with variant calling; both of which use sets of rules that follow ACMG criteria: Franklin (https://franklin.genoox.com) and Varsome (https://varsome.com), both last accessed on 28 April 2023. Variants found were compared with variations listed in ClinVar (https://www.ncbi.nlm.nih.gov/clinvar/ accessed on 28 April 2023).

This study was performed in accordance with the Declaration of Helsinki and the protection of patient identity and was approved by the Research Ethics Committee of the Federal University of São Paulo (protocol number 5.113.810). Written informed consent was obtained whenever it was necessary to perform molecular tests. When DNA samples were collected for molecular tests, all patients and/or their legal guardians provided written informed consent for the use of the personal medical data for scientific purposes and publication.

## 3. Results and Discussion

We identified 46 patients with clinical diagnosis of ACHM. Forty-two patients presented biallelic pathogenic variants in the *CNGA3* and *CNGB3* genes, while four patients presented biallelic pathogenic variants in the *PDE6C* gene. In our cohort, we did not find any ACHM patients related to the other described genes (*PDE6H*, *GNAT2*, or *ATF6*).

Table 1 summarizes the genotype identified in the affected individuals with *CNGA3* variants, while Table 2 shows the same results for *CNGB3* variants. Both tables show the classification according to the ACMG guidelines.

In this Brazilian sample of 42 patients from 38 families with ACHM related to biallelic variants in CNG genes (19 patients in *CNGA3* and 23 patients in *CNGB3*), 20 pathogenic variants in *CNGA3* gene and 11 pathogenic variants in *CNGB3* gene were identified, including one novel variant in *CNGB3* gene. The pathogenicity of this novel variant was “likely pathogenic” according to the ACMG classification.

In this cohort, among *CNGA3* variants, sixteen were missense variants (80%), three were nonsense (15%), and one was an initiation codon variant (5%). *CNGA3* pathogenic variants were found in 46% of patients. These findings are in line with previously conducted studies in the literature [6,12], where the most prevalent variants were found to be missense followed by nonsense.

Considering *CNGB3* variants, three were frameshift (27%), four were nonsense (36%), three were splice-site variants (27%), and one was an initiation codon variant (9%). The most common *CNGB3* variant found in this cohort was the deletion c.1148delC (p.Thr383Ilefs*13), resulting in a frameshift and premature stop codon most prevalent in homozygosity; this accounted for 45% (11 patients) of all *CNGB3*-linked genotypes, while six patients (25%) presented this variant in at least one allele in heterozygosis. One nonsense novel variant (c.1893T > A; p.Tyr631*) is described here for the first time. This variant meets a very strong (PVS1) and a supporting (PM2) ACMG criteria, with extremely low frequency in gnomAD databases. This sequence change creates a premature stop signal in the *CNGB3* gene. It is expected to result in an absent or disrupted protein product. Loss of function variants in *CNGB3* are known to be a mechanism of disease; 185 pathogenic null variants were reported in ClinVar for this gene across 18 different exons. This variant was classified as deleterious by three pathogenicity predictors (MutationTaster, DANN and BayesDel), was classified as “likely pathogenic” by the reporting lab and was absent in ClinVar.

Table 3 resumes variant data, showing the allele count in this cohort and the total allele frequency from all populations in gnomAD.

The prevalence of *CNGA3* or *CNGB3* variants varies globally [9,10,11]. Mayer et al. evaluated *CNGB3* pathogenic variants in 485 independent families with ACHM, mainly of Western Europe and North America descent [10]. The c.1148delC variant was by far the most common variant in that cohort, accounting for 66% of all *CNGB3*-linked ACHM alleles. It was demonstrated that the high prevalence of this variant was due to a founder effect, and the presence of this variant in European population is most likely due to a single mutation event [10].

The cone CNG channel is composed of three CNGA3 and one CNGB3 subunits located exclusively in the plasma membrane of the outer segment of cone photoreceptors [15]. To date, more than 230 pathogenic variants in *CNGA3* [12] and around 200 pathogenic variants in *CNGB3* [10] have been found to cause inherited ACHM in humans. All known disease-causing variants are inherited in an autosomal recessive manner, and only homozygous or compound heterozygous patients show the typical symptoms of ACHM.

Table 1 and Table 2 summarize BCVA and CS exams. BCVA ranged from 20/100 to 5/400. The only patient presenting CF in one eye (P31.1) had been submitted to vitrectomy surgery in the right eye due to retinal detachment after contuse trauma. When available, CS ranged from 0.55 to 1.65 logCS. These findings did not show any correlation with age. Another patient presented reduced visual acuity and CS (P15.2) with a severe phenotype that differs from other patients, including his affected sister (P15.1).

One clinical trial with ACHM associated with the *CNGA3* gene has published 1-year [16] and 3-year follow-ups [17], showing improvements in secondary endpoints in visual acuity and contrast sensitivity compared to baseline data.

**Figure 1:**
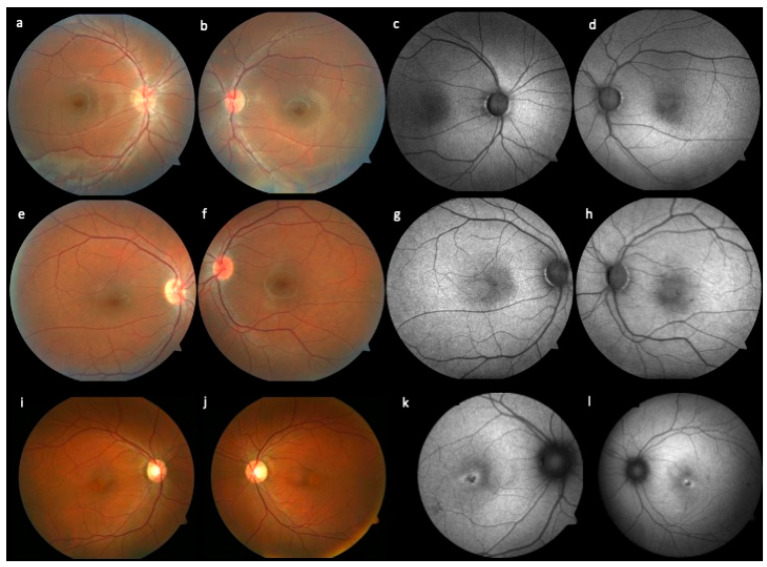
(**a**–**l**)**.** Representative fundus images from three patients with different findings. Color photos (**a**,**b**) and FAF (**c**,**d**) of P24.2, presenting normal fundus appearance and normal fundus autofluorescence. P24.1 photos reveal normal fundus appearance (**e**,**f**) and slight changes of the perifoveal autofluorescence (**g**,**h**). In P22.1, central atrophy (**i**,**j**) and fovea hypoautofluorescence atrophy surrounded by a hyperautofluorescence ring (**k**,**l**) can be seen.

Fundoscopy findings varied from normal fundus appearance to atrophy in the foveal area. Correlating to color fundus photos, FAF presented different findings, varying from a normal fundus autofluorescence to a reduced autofluorescence with subtle hyperautofluorescence ring around the fovea. Figure 1 exemplifies the retinal findings.

**Figure 2:**
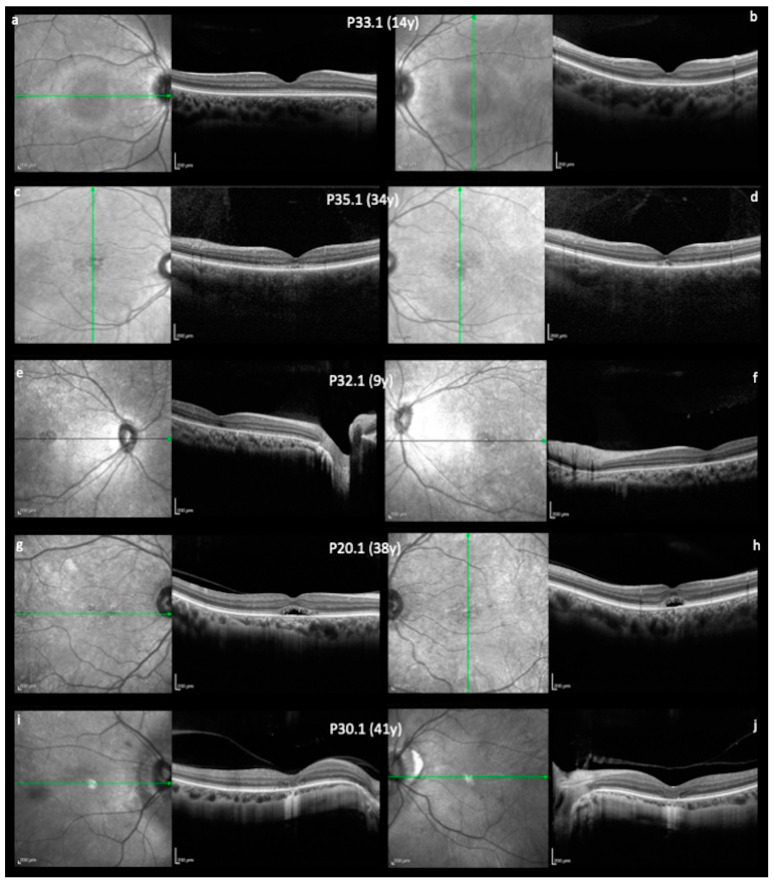
(**a**–**j**) Macular spectral domain OCT of five patients with different stages and their correspondent ages. Green line corresponds to horizontal and vertical foveal scans. (**a**,**b**) P33.1 (14 years old) with normal retinal layers and continuous ellipsoid zone. (**c**,**d**) P35.1 (34 years old), presenting disrupted ellipsoid band. (**e**,**f**) P32.1 (9 years old) had inner segment ellipsoid loss. (**g**,**h**) P20.1 (38 years old) had hyporeflective zone. (**i**,**j**) P30.1 (41 years old) presented outer retinal atrophy, including RPE loss.

On morphologic exams, OCT shows varying degrees of foveal abnormalities in the inner segment ellipsoid zone. Representative OCT images are shown in Figure 2. Because certain patient had poor fixation, it was not possible to obtain good horizontal scans for all patients. The severity of degeneration shown on the OCT was graded in different stages.

Several clinical studies have investigated outer retinal and foveal morphology in detail by using high resolution OCT in ACHM [18]. The macular appearance in OCT can either show normal anatomy architecture or variable degrees of disruption of the photoreceptor layers and loss of RPE. Previously published cross-sectional studies have described conflicting findings with respect to the age dependency of progression in OCT [18]. Aboshiba et al. suggested that retinal structure alterations in ACHM may be slowly progressive and subtle in most patients and may not be correlated with age or genotype [19]. Triantafylla et al. showed longitudinal changes in foveal structure, mainly in children, though in adults with ACHM as well, over a long follow-up period [20]. Four stages of morphological degenerative changes have been described in ACHM: preserved inner segment ellipsoid, disrupted inner segment ellipsoid, inner segment ellipsoid loss, and inner segment and RPE loss [21]. However, whether morphological changes over time follow the proposed four-stage linear pattern needs to be confirmed through long-term studies.

Great variability in OCT findings was observed in our patients (Figure 2). Considering disease onset at birth or early childhood, we did not find a consistent correlation with age or disease stage in OCT foveal morphology. There were varying degrees of abnormalities in the inner segment ellipsoid observed in both young and elderly patients. This suggests that progression may not be age dependent.

Limitations of our study include its retrospective nature and the consequent fact that not all data were available for every patient. In addition, data were acquired using various methods and protocols. Finally, segregation data and detailed clinical information were limited.

## 4. Conclusions

This paper has presented a considerable cohort of patients with ACHM related to the *CNGA3* and *CNGB3* genes. The current development of gene therapy for ACHM requires characterization of these patients in detail in order to better understand disease evolution.

## Figures and Tables

**Table 1:** Clinical and genetic results from patients with *CNGA3* variants.

Family	ID	Age of Onset	Sings/Symptoms Onset	Current BCVA	CS (logCS)	Gene	Nucleotide and Protein Changes	Zygosity	ACMG Classification
				RE; LE	RE; LE				
1	1.1	9 months	Nystagmus; photophobia	20/100; 20/100	N/A	CNGA3	c.67C > T (p.Arg23*) c.1687C > T (p.Arg563Cys)	heterozygous heterozygous	pathogenic pathogenic
2	2.1	5 months	Nystagmus; photophobia	20/400; 20/400	1.35; 1.50	CNGA3	c.1775C > T (p.Pro592Leu) c.829C > T (p.Arg277Cys)	heterozygous heterozygous	pathogenic pathogenic
3	3.1	N/A	Photophobia, color blindness	N/A	N/A	CNGA3	c.1717T > C (p.Tyr573His)	homozygous	pathogenic
4	4.1	3 months	Nystagmus; photophobia	20/125; 20/125	1.50; 1.45	CNGA3	c.572G > A (p.Cys191Tyr) c.811C > G (p.Pro271Ala)	heterozygous heterozygous	pathogenic likely pathogenic
5	5.1	3 months	Nystagmus; photophobia	5/400; 5/400	N/A	CNGA3	c.1775C > T (p.Pro592Leu)	homozygous	pathogenic
6	6.1	Since birth	Nystagmus	20/70; 20/70	1.60; 1.65	CNGA3	c.1669G > A (p.Gly557Arg)	homozygous	likely pathogenic
7	7.1	Childhood	Low central vision	20/50; 20/150	N/A	CNGA3	c.1669G > A (p.Gly557Arg) c.1981C > A (p.Arg661Ser)	heterozygous heterozygous	likely pathogenic pathogenic
8	8.1	Since birth	Nystagmus; photophobia	20/200; 20/200	1.30; 1.35	CNGA3	c.1585G > A (p.Val529Met) c.1319G > A (p.Trp440*)	heterozygous heterozygous	pathogenic pathogenic
9	9.1	3 months	Nystagmus; photophobia	20/200; 20/160	1.30; 1.25	CNGA3	c.1669G > A (p.Gly557Arg) c.967G > C (p.Ala323Pro)	heterozygous heterozygous	likely pathogenic likely pathogenic
10	10.1	Childhood	Photophobia; color blindness	20/100; 20/100	1.30; 1.00	CNGA3	c.1279C > T (p.Arg427Cys) c.1717T > C (p.Tyr573His)	heterozygous heterozygous	pathogenic pathogenic
11	11.1	2 months	Nystagmus	N/A	N/A	CNGA3	c.1641C > A (p.Phe547Leu)	homozygous	pathogenic
12	12.1	4 months	Nystagmus; photophobia	20/125; 20/125	N/A	CNGA3	c.1981C > A (p.Arg661Ser) c.778G > A (p.Asp260Asn)	heterozygous heterozygous	pathogenic pathogenic
13	13.1	Since birth	Nystagmus	20/400; 20/400	1.15; 1.30	CNGA3	c.1495C > T (p.Arg499*) c.572G > A (p.Cys191Tyr)	heterozygous heterozygous	pathogenic pathogenic
14	14.1	2 years	Nystagmus; photophobia	20/150; 20/150	N/A	CNGA3	c.2T > G (p.Met1?) c.1306C > T (p.Arg436Trp)	heterozygous heterozygous	likely pathogenic pathogenic
15	15.1	Since birth	Nystagmus	20/160; 20/250	1.60; 1.45	CNGA3	c.1279C > T (p.Arg427Cys) c.1495C > T (p.Arg499*)	heterozygous heterozygous	pathogenic pathogenic
15	15.2	Since birth	Nystagmus; photophobia	HM; 20/640	0.0; 0.15	CNGA3	c.1279C > T (p.Arg427Cys) c.1495C > T (p.Arg499*)	heterozygous heterozygous	pathogenic pathogenic
16	16.1	Since birth	Nystagmus; photophobia	N/A	N/A	CNGA3	c.1201T > C (p.Ser401Pro)	homozygous	likely pathogenic
17	17.1	1 year	Nystagmus	20/80; 20/100	1.15; 1.35	CNGA3	c.1279C > T (p.Arg427Cys) c.1495C > T (p.Arg499*)	heterozygous heterozygous	pathogenic pathogenic
18	18.1	Since birth	Photophobia	20/200; 20/200	1.35; 1.35	CNGA3	c.1585G > A (p.Val529Met) c.847C > T (p.Arg238Trp)	heterozygous heterozygous	pathogenic pathogenic

BCVA: Best corrected visual acuity; RE: right eye; LE: left eye; N/A: not available; HM: hand motion; CF: count fingers.

**Table 2:** Clinical and genetic results from patients with *CNGB3* variants.

Family	ID	Age of Onset	Sings/Symptoms Onset	Current BCVA	CS (logCS)	Gene	Nucleotide and Protein Changes	Zygosity	ACMG Classification
19	19.1	3 months	Nystagmus; photophobia	10/400; 10/400	N/A	CNGB3	c.1148delC (p.Thr383Ilefs*13)	homozygous	pathogenic
20	20.1	Since birth	Nystagmus; photophobia	20/200; 20/150	1.60; 1.45	CNGB3	c.1148delC (p.Thr383Ilefs*13)	homozygous	pathogenic
21	21.1	Since birth	Nystagmus; photophobia	20/400; 20/400	N/A	CNGB3	c.1148delC (p.Thr383Ilefs*13) c.1285delT (p.Ser429Leufs*9)	heterozygous heterozygous	pathogenic pathogenic
22	22.1	Since birth	Photophobia	20/200; 20/400	1.35; 1.35	CNGB3	c.1148delC (p.Thr383Ilefs*13) c.2T > C (p.Met1?)	heterozygous heterozygous	pathogenic pathogenic
23	23.1	Since birth	Photophobia	20/400; 20/200	1.15; 1.20	CNGB3	c.1148delC (p.Thr383Ilefs*13) c.903 + 1G > A (p.?)	heterozygous heterozygous	pathogenic pathogenic
24	24.1	Since birth	Nystagmus	20/250; 20/250	1.30; 1.60	CNGB3	c.1148delC (p.Thr383Ilefs*13)	homozygous	pathogenic
24	24.2	Since birth	Nystagmus	20/160; 20/250	1.45; 1.10	CNGB3	c.1148delC (p.Thr383Ilefs*13)	homozygous	pathogenic
24	24.3	Since birth	Nystagmus	20/250; 20/250	1.30; 1.35	CNGB3	c.1148delC (p.Thr383Ilefs*13)	homozygous	pathogenic
25	25.1	2 years	Nystagmus	N/A	N/A	CNGB3	c.1148delC (p.Thr383Ilefs*13)	homozygous	pathogenic
25	25.2	3 months	Nystagmus	N/A	N/A	CNGB3	c.1148delC (p.Thr383Ilefs*13)	homozygous	pathogenic
26	26.1	Since birth	Nystagmus; photophobia	20/150; 20/150	1.35; 1.20	CNGB3	c.566G > A (p.Trp189*)	homozygous	pathogenic
27	27.1	Since birth	Nystagmus; photophobia	20/100; 20/100	N/A	CNGB3	c.1148delC (p.Thr383Ilefs*13)	homozygous	pathogenic
28	28.1	Since birth	Nystagmus; photophobia	20/160; 20/160	1.40; 1.30	CNGB3	c.1148delC (p.Thr383Ilefs*13) c.566G > A (p.Trp189*)	heterozygous heterozygous	pathogenic pathogenic
29	29.1	N/A	N/A	20/160; 20/160	1.20; 1.10	CNGB3	c.852 + 1G > T (p.?)	homozygous	pathogenic
30	30.1	N/A	Nystagmus; photophobia	CF; 20/80	N/A	CNGB3	c.446_447insT (p.Lys149Asnfs*30)	homozygous	pathogenic
31	31.1	6 months	Photophobia	20/400; 20/320	0.55; 1.45	CNGB3	c.1432C > T (p.Arg478*)	homozygous	pathogenic
32	32.1	Since birth	Nystagmus; photophobia	20/160; 20/160	1.50; 1.55	CNGB3	c.1148delC (p.Thr383Ilefs*13)	homozygous	pathogenic
33	33.1	4 months	Nystagmus	20/160; 20/200	1.30; 1.30	CNGB3	c.1810C > T (p.Arg604*)	homozygous	pathogenic
34	34.1	Since birth	Photophobia	20/125; 20/200	1.40; 1.20	CNGB3	c.1148delC (p.Thr383Ilefs*13) c.991-3T > G (p.?)	heterozygous heterozygous	pathogenic likely pathogenic
35	35.1	2 months	Nystagmus	N/A	N/A	CNGB3	c.566G > A (p.Trp189*)	homozygous	pathogenic
36	36.1	Since birth	Nystagmus, photophobia	20/125; 20/100	N/A	CNGB3	c.1148delC (p.Thr383Ilefs*13) c.1893T > A (p.Tyr631*)	heterozygous heterozygous	pathogenic likely pathogenic (novel)
37	37.1	N/A	Photophobia; color blindness	20/400; 20/400	N/A	CNGB3	c.1148delC (p.Thr383Ilefs*13)	homozygous	pathogenic
38	38.1	4 months	Nystagmus	20/250; 20/200	1.30; 1.35	CNGB3	c.1148delC (p.Thr383Ilefs*13)	homozygous	pathogenic

**Table 3:** Variant data with allele count in this cohort and total allele frequency from all populations of the gnomAD database (accessed on 28 April 2023).

Causative Gene	Transcript	Nucleotide Change	Consequence	Patients Evaluated		gnomAD Allele Frequency (%)
				Allele Count	Number of Homozygotes	
*CNGA3*	NM_001298.3	c.67C > T	(p.Arg23*)	1	0	0.003540
*CNGA3*	NM_001298.3	c.1687C > T	(p.Arg563Cys)	1	0	0.002122
*CNGA3*	NM_001298.2	c.1775C > T	(p.Pro592Leu)	3	1	0.0003980
*CNGA3*	NM_001298.2	c.829C > T	(p.Arg277Cys)	1	0	0.009548
*CNGA3*	NM_001298.2	c.1717T > C	(p.Tyr573His)	3	1	0.003187
*CNGA3*	NM_001298.2	c.572G > A	(p.Cys191Tyr)	2	0	0.002121
*CNGA3*	NM_001298.2	c.811C > G	(p.Pro271Ala)	1	0	0.01202
*CNGA3*	NM_001298.2	c.1669G > A	(p.Gly557Arg)	4	1	0.01415
*CNGA3*	NM_001298.2	c.1981C > A	(p.Arg661Ser)	2	0	0.03084
*CNGA3*	NM_001298.2	c.1585G > A	(p.Val529Met)	2	0	0.006726
*CNGA3*	NM_001298.2	c.1319G > A	(p.Trp440*)	1	0	0.0003986
*CNGA3*	NM_001298.2	c.967G > C	(p.Ala323Pro)	1	0	0.009544
*CNGA3*	NM_001298.2	c.1279C > T	(p.Arg427Cys)	4	0	0.03902
*CNGA3*	NM_001298.3	c.1641C > A	(p.Phe547Leu)	2	1	0.01592
*CNGA3*	NM_001298.2	c.778G > A	(p.Asp260Asn)	1	0	0.003182
*CNGA3*	NM_001298.2	c.1495C > T	(p.Arg499*)	4	0	0.001063
*CNGA3*	NM_001298.2	c.2T > G	(p.Met1?)	1	0	-
*CNGA3*	NM_001298.3	c.1306C > T	(p.Arg436Trp)	1	0	0.009574
*CNGA3*	NM_001298.3	c.1201T > C	(p.Ser401Pro)	1	0	0.0003995
*CNGA3*	NM_001298.2	c.847C > T	(p.Arg238Trp)	1	0	0.009948
*CNGB3*	NM_019098.4	c.1148delC	(p.Thr383Ilefs*13)	28	11	0.1750
*CNGB3*	NM_019098.5	c.1285delT	(p.Ser429Leufs*9)	1	0	0.000399
*CNGB3*	NM_019098.5	c.2T > C	(p.Met1?)	1	0	-
*CNGB3*	NM_019098.4	c.903 + 1G > A	(p.?)	1	0	-
*CNGB3*	NM_019098.4	c.566G > A	(p.Trp189*)	5	2	0.0003977
*CNGB3*	NM_019098.4	c.852 + 1G > T	(p.?)	2	1	-
*CNGB3*	NM_019098.4	c.446_447insT	(p.Lys149Asnfs*30)	2	1	0.0003977
*CNGB3*	NM_019098.4	c.1432C > T	(p.Arg478*)	2	1	0.001991
*CNGB3*	NM_019098.5	c.1810C > T	(p.Arg604*)	2	1	0.0007969
*CNGB3*	NM_019098.4	c.991-3T > G	(p.?)	1	0	0.001338
*CNGB3*	NM_019098.5	c.1893T > A	(p.Tyr631*)	1	0	-

## Data Availability

Not applicable.

## Supplementary Materials

The following supporting information can be downloaded at: https://www.mdpi.com/article/10.3390/genes14061296/s1, Table S1: Genes included in the NGS panels used.
