# Supplementary material for: Molecular and Clinical Characterization of CNGA3 and CNGB3 Genes in Brazilian Patients Affected with Achromatopsia
Source: Genes (Basel). 2023 Jun 20;14(6):1296. doi: 10.3390/genes14061296 (PMC10298554; doi:10.3390/genes14061296)
Supplement: Supplementary file 1 [file genes-14-01296-s001.zip › genes-2403614-supplementary.pdf]

Supplemental Table S1. Genes included in the NGS panels used.

IRD NGS panel (224 IRD-associated genes)	IRD NGS panel (330 IRD-associated genes)
ABCA4, ABCB5, ABCC6, ABHD12, ACO2, ADAM9, ADAMTS9, ADGRV1, AFG3L2, AGBL5, AHI1, AHR, AIPL1, ALMS1, ALPK1, AMACR, ARFGAP2, ARHGEF18, ARL13B, ARL2BP, ARL3, ARL6, ARMC9, ARSG, ASRGL1, ATF6, B9D1, B9D2, BBIP1, BBS1, BBS10, BBS12, BBS2, BBS4, BBS5, BBS7, BBS9, BEST1, C1QTNF5, CA4, CABP4, CACNA1F, CACNA2D4, CASK, CC2D2A, CDH23, CDH3, CDHR1, CEP120, CEP162, CEP164, CEP250, CEP290, CEP41, CEP78, CERKL, CFAP20, CFAP410, CFAP418, CFH, CHM, CISD2, CLCC1, CLEC3B, CLN3, CLN5, CLN6, CLN8, CLRN1, CNGA1, CNGA3, CNGB1, CNGB3, CNNM4, COL18A1, COL9A3, CPLANE1, CRB1, CROCC, CRX, CSPP1, CTNNA1, CTNNB1, CTSD, CTSF, CWC27, CYP4V2, DHDDS, DHX38, DNAJC17, DNAJC5, DNM1L, DRAM2, DYNC2H1, EFEMP1, ELOVL4, ESPN, EXOSC2, EYS, FAM161A, FLVCR1, FRMD7, FSCN2, FZD4, GDF6, GJB2, GNAT1, GNAT2, GNPTG, GPR143,	ABCA4, ABCC6, ABHD12, ACBD5, ACO2, ADAM9, ADAMTS18, ADAMTSL4, ADGRA3, ADGRV1, ADIPOR1, AGBL5, AHI1, AHR, AIPL1, ALMS1, ARHGEF18, ARL13B, ARL2BP, ARL3, ARL6, ARMC9, ARSG, ASRGL1, ATF6, ATOH7, B9D1, BBIP1, BBS1, BBS10, BBS12, BBS2, BBS4, BBS5, BBS7, BBS9, BEST1, C10ORF11, C12ORF65, C1QTNF5, C8ORF37, CA4, CABP4, CACNA1F, CACNA2D4, CAPN5, CC2D2A, CCT2, CDH23, CDH3, CDHR1, CEP164, CEP19, CEP250, CEP290, CEP41, CEP78, CEP83, CERKL, CFAP410, CHM, CIB2, CISD2, CLCC1, CLN2 (TPP1), CLN3, CLN5, CLN6, CLN8, CLRN1, CLUAP1, CNGA1, CNGA3, CNGB1, CNGB3, CNNM4, COL11A1, COL11A2, COL18A1, COL2A1, COL9A1, COL9A2, COL9A3, CPLANE1, CRB1, CRX, CSPP1, CTNNA1, CTSD, CWC27, CYP4V2, DHDDS, DHX32, DHX38, DNAJC17, DRAM2, DSCAML1, DTHD1, EFEMP1, ELOVL4, EMC1, ERCC6, EXOSC2, EYS, FAM161A, FBLN5, FLVCR1, FRMD7, FSCN2, FZD4, GDF6, GNAT1, GNAT2, GNB3, GNPTG, GNS, GPR143, GPR179, GPR45, GRM6, GRN, GUCA1A, GUCA1B, GUCY2D, HARS, HGSNAT, HK1, HMCN1, HMX1,

GPR179, GRK1, GRM6, GRN, GUCA1A, GUCA1B, GUCY2D, HGSNAT, HK1, HKDC1, HMCN1, HMX1, IDH3A, IDH3B, IFT140, IFT172, IFT27, IFT43, IFT74, IFT88, IGFBP7, IMPDH1, IMPG1, IMPG2, INPP5E, IQCB1, IRX5, ITM2B, KCNJ13, KCNV2, KCTD7, KIAA0586, KIAA0753, KIAA1549, KIF3B, KIF7, KIZ, KLHL7, LAMA1, LCA5, LRAT, LRIT3, LRP5, LZTFL1, MAK, MAPKAPK3, MERTK, MFN2, MFRP, MFSD8, MIR204, MKKS, MKS1, MMACHC, MVK, MYO7A, NDP, NEK2, NEUROD1, NMNAT1, NPHP1, NPHP3, NPHP4, NR2E3, NR2F1, NRL, NUMB, NYX, OAT, OFD1, OPA1, OPA3, OPN1LW, OTX2, PANK2, PAX6, PCARE, PCDH15, PDE6A, PDE6B, PDE6C, PDE6D, PDE6G, PDE6H, PDZD7, PEX1, PEX10, PEX11B, PEX12, PEX13, PEX14, PEX16, PEX19, PEX2, PEX26, PEX3, PEX5, PEX6, PEX7, PHYH, PIBF1, PITPNM3, PLK4, PNPLA6, POC1B, POC5, PPP2R3C, PPP2R5E, PPT1, PRCD, PROM1, PRPF3, PRPF31, PRPF4, PRPF6, PRPF8, PRPH2, PRPS1, PYGM, RAB28, RAX2, RBP3, RBP4, RCBTB1, RD3, RDH11, RDH12,	IDH3A, IDH3B, IFT140, IFT172, IFT27, IFT43, IFT74, IFT80, IFT81, IFT88, IMPDH1, IMPG1, IMPG2, INPP5E, INVS, IQCB1, ITM2B, JAG1, KCNJ13, KCNV2, KIAA0586, KIAA1549, KIF11, KIF7, KIZ, KLHL7, LCA5, LRAT, LRIT3, LRP2, LRP5, LYST, LZTFL1, MAK, MAPKAPK3, MERTK, MFN2, MFRP, MFSD8, MIR204, MKKS, MKS1, MPDZ, MTPAP, MTTP, MYO7A, NAGLU, NBAS, NDP, NEK2, NEUROD1, NMNAT1, NPHP1, NPHP3, NPHP4, NR2E3, NR2F1, NRL, NYX, OAT, OCA2, OFD1, OPA1, OPA3, OPN1SW, OR2W3, OTX2, P3H2, PAX2, PAX6, PCARE, PCDH15, PCYT1A, PDE6A, PDE6B, PDE6C, PDE6D, PDE6G, PDE6H, PDZD7, PEX1, PEX10, PEX11B, PEX12, PEX13, PEX14, PEX16, PEX19, PEX2, PEX26, PEX3, PEX5, PEX6, PEX7, PHYH, PITPNM3, PLA2G5, PLK4, PNPLA6, POC1B, POC5, POMGNT1, PPT1, PRCD, PRDM13, PROM1, PRPF3, PRPF31, PRPF4, PRPF6, PRPF8, PRPH2, PRPS1, RAB28, RAX2, RBP1, RBP3, RBP4, RCBTB1, RD3, RDH11, RDH12, RDH5, REEP6, RGR, RGS9, RGS9BP, RHO, RIMS1, RLBP1, ROM1, RP1, RP1L1, RP2, RP9, RPE65, RPGR, RPGR (ORF15), RPGRIP1, RPGRIP1L, RS1, RTN4IP1, SAG, SAMD11, SCLT1, SDCCAG8, SEMA4A, SGSH, SIX6, SLC24A1, SLC24A5, SLC45A2, SLC7A14, SNRNP200, SPATA7, SPP2,

RDH5, REEP6, RGR, RGS9, RGS9BP, RHO, RIMS1, RLBP1, ROM1, RP1, RP1L1, RP2, RP9, RPE65, RPGR, RPGRIP1, RPGRIP1L, RS1, RTN4IP1, SAG, SCAPER, SCLT1, SDCCAG8, SEMA4A, SIX6, SLC24A1, SLC30A7, SLC39A12, SLC66A1, SLC6A6, SLC7A14, SNRNP200, SPATA7, SSBP1, STX3, SUFU, TCTN1, TCTN2, TCTN3, TEAD1, TIMM8A, TIMP3, TLCD3B, TMEM107, TMEM126A, TMEM138, TMEM216, TMEM218, TMEM231, TMEM237, TMEM67, TOGARAM1, TOPORS, TPP1, TRAPPC3, TRIM32, TRPM1, TSPAN12, TTC21B, TTC8, TTLL5, TUB, TUBB4B, TUBGCP4, TUBGCP6, TULP1, TXNDC15, TYR, UNC119, USH1C, USH1G, USH2A, USO1, USP45, VPS13B, WDPCP, WDR19, WFS1, WHRN, YME1L1, ZNF408, ZNF423, ZNF513	TCTN1, TCTN2, TCTN3, TEAD1, TIMM8A, TIMP3, TMED7, TMEM107, TMEM126A, TMEM138, TMEM216, TMEM231, TMEM237, TMEM67, TOPORS, TRAF3IP1, TREX1, TRIM32, TRNT1, TRPM1, TSPAN12, TTC21B, TTC8, TTLL5, TTPA, TUB, TUBGCP4, TUBGCP6, TULP1, TYR, TYRP1, UNC119, USH1C, USH1G, USH2A, VCAN, VPS13B, WDPCP, WDR19, WDR34, WFS1, WHRN, ZNF408, ZNF423, ZNF513
